# Duplex loop-mediated isothermal amplification assay for simultaneous detection of human and human male DNA

**DOI:** 10.1186/s13104-023-06464-2

**Published:** 2023-08-22

**Authors:** Seiji Kubo, Hideki Niimi, Isao Kitajima

**Affiliations:** 1 Department of Clinical Laboratory and Molecular Pathology, Faculty of Medicine, Academic Assembly, University of Toyama, 2630 Sugitani, Toyama, 930-0194 Japan; 2 Forensic Science Laboratory, Ishikawa Prefectural Police Headquarters, 1-1 Kuratsuki, Kanazawa, 920-8553 Japan; 3 Administrative Office, University of Toyama, 3190 Gofuku, Toyama, 930-8555 Japan

**Keywords:** Loop-mediated isothermal amplification, Duplex assay, Human DNA, Human male DNA

## Abstract

**Objective:**

Screening of human and human male DNA is necessary for forensic DNA analyses. Although quantitative real-time PCR (qPCR) is commonly used for detecting and quantifying these DNA targets, its use as a screening tool is time-consuming and labor-intensive. To streamline and simplify the screening process, we aimed to develop a duplex loop-mediated isothermal amplification (LAMP) assay capable of simultaneously detecting human and human male DNA in a single tube. We assessed the duplex LAMP assay for forensic application.

**Results:**

For our duplex LAMP assay, we have utilized two fluorescent probes with HEX and FAM fluorophores to specifically detect human and human male DNA, respectively. The HEX (human target) signal was detected from both the male and female DNA samples, and the FAM (male target) signal was detected from only the male DNA sample. This assay has a sensitivity of 10–1 pg of DNA for both targets. Additionally, we successfully detected the two targets in the DNA samples extracted from forensically relevant body fluids, including blood, saliva, semen, and vaginal secretions.

**Supplementary Information:**

The online version contains supplementary material available at 10.1186/s13104-023-06464-2.

## Introduction

Forensic samples found at crime scenes often contain only low-level DNA, no DNA at all, or DNA irrelevant to the crime. Thus, screening of forensic samples is necessary for DNA typing, i.e., short tandem repeat (STR) analysis. The presence of human or human male DNA is the criteria for proceeding with the downstream STR analysis. In general, quantitative real-time PCR (qPCR) is used to detect human and human male DNA [1, 2]. Although qPCR is a reliable quantification method, its use as a screening tool takes time and effort.

A loop-mediated isothermal amplification (LAMP) assay is a rapid and simple method for detecting nucleic acids [3, 4]. The reaction can be completed within 20–60 min under the isothermal conditions of 60–65 ℃. Thus, we hypothesized that its rapidity and simplicity make it suitable for screening human and human male DNA. Previous studies have reported the LAMP assays targeting human DNA for forensic use [5–7]. Another study has reported a male DNA-specific LAMP assay that can detect as low as 1 pg of male DNA within 20 min [8]. However, unlike the multiplex qPCR assay [1, 2], the reported LAMP assays can detect only one target per assay. To our knowledge, duplex LAMP assays have yet to be developed for forensic application.

**Fig. 1:**
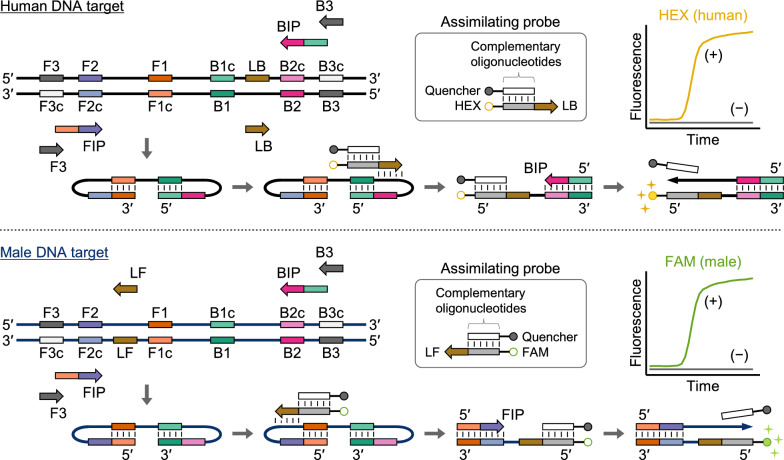
Schematic representation of our duplex LAMP assay

We developed a fluorescence-based duplex LAMP assay to simultaneously detect human and human male DNA, using an assimilating probe [9, 10]. The probe comprises a fluorescent strand (5′-fluorophore-oligonucleotides-loop primer-3′) and a quenching strand (5′-complementary oligonucleotides-quencher-3′) (Fig. 1). Hybridization of the oligonucleotide region brings the fluorophore and quencher into proximity, thereby causing fluorescence quenching. Upon encountering the LAMP intermediate, the assimilating probe acts as a primer and extends to the inner primer region (FIP or BIP). Subsequently, the quenching strand is displaced by a newly synthesized strand from the inner primer (FIP or BIP), leading to fluorescence emission. We assessed the feasibility of our duplex LAMP assay for forensic application.

## Materials and methods

### Control DNA

Male and female genomic DNA (G1471 and G1521, respectively; Promega, Madison, WI) were used as the control DNA samples. Serial dilutions of the control DNA samples (100 ng–1 pg) were used for the sensitivity test. Mixed control DNA samples prepared in male:female ratio ranging from 1:1 to 1:10,000 (male DNA kept at 10 pg) were used for the mixture analysis.

### Body fluids

Blood, saliva, semen, and vaginal secretions were collected from healthy volunteers. Male blood (0.25 µL), male saliva (0.25 µL), semen (0.25 µL), female blood (50 µL), female saliva (50 µL), and half of a vaginal swab head were used for the specificity test (3 donors each).

### DNA extraction

DNA was extracted from body fluid samples using the EZ1 DNA Investigator Kit (QIAGEN, Venlo, The Netherlands) according to the manufacturer’s protocol. Blood, saliva, and vaginal swab samples were lysed in 290 µL of G2 buffer (QIAGEN) and 10 µL of Proteinase K (QIAGEN). Semen samples were lysed in 270 µL of G2 buffer, 10 µL of Proteinase K, and 20 µL of 1 M dithiothreitol (Fujifilm Wako Pure Chemical Corp., Osaka, Japan). Then, the extracted DNA was purified using the EZ1 Advanced XL instrument (QIAGEN) with the following setting: Trace protocol with 50 µL of TE buffer elution.

### Primer and probe

Multicopy locus of the autosomal DNA (GenBank accession number: M13882) was selected as the target of human DNA [5]. Multicopy locus of the Y chromosome DNA (GenBank accession number: AF522078) was selected as the target of human male DNA [8, 11–14]. The LAMP primers were designed following previous reports [5, 8]. The assimilating probes were designed based on previous studies [9, 10]; the HEX-labeled probe was used for total human DNA, and the FAM-labeled probe was used for human male DNA (Fig. 1). Primers and probes were purchased from Eurofins Genomics K.K. (Tokyo, Japan). Sequences are listed in Additional file 2: Table S1. HD-primer mix (for human DNA), Y-primer mix (for male DNA), and probe mix (for both targets) were prepared at 10 × concentration. Final (1 ×) concentrations are shown in Additional file 2: Table S1.

### Duplex LAMP assay

The duplex LAMP assay was performed using the LAMP-FLP kit (Nippon Gene, Tokyo, Japan). The 25 µL of reaction mixture contained 1 × reaction buffer, 1.4 µL of dNTPs mixture, 1 × HD-primer mix, 1 × Y-primer mix, 1 × probe mix, 1 µL of polymerase, and 2 µL of the DNA template. The reaction was conducted at 65 ℃ for 60 min using the QuantStudio^™^ 5 Real-Time PCR System (Thermo Fisher Scientific, Waltham, MA). Fluorescence was measured every 30 s in the FAM (excitation, 470 ± 15 nm; emission, 520 ± 15 nm) and VIC (excitation, 520 ± 10 nm; emission, 558 ± 12 nm; for HEX) channels. The data was analyzed using QuantStudio^™^ Design & Analysis Software version 1.5.1 (Thermo Fisher Scientific) with the default settings (the automatic baseline and threshold settings). The quantification cycle (Cq) was calculated automatically. The obtained Cq value was defined as the threshold time (1 min = 1 cycle).

## Results

### Assay confirmation

**Fig. 2:**
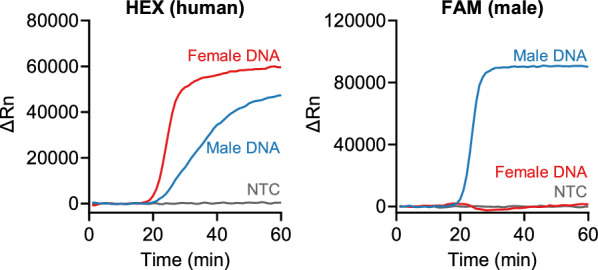
Confirmation of the duplex LAMP assay. Representative amplification plots of the fluorescence analysis. Male DNA, 2 ng; Female DNA, 20 ng; NTC, no-template control

We performed the duplex LAMP assay on the male DNA, female DNA, and no-template control (NTC) samples. Our assay detected the HEX (human target) signal from the male and female DNA samples, and the FAM (male target) signal from only the male DNA sample (Fig. 2). No signals were detected from the NTC sample. Therefore, we confirmed the success of our duplex LAMP assay.

### Sensitivity

**Fig. 3:**
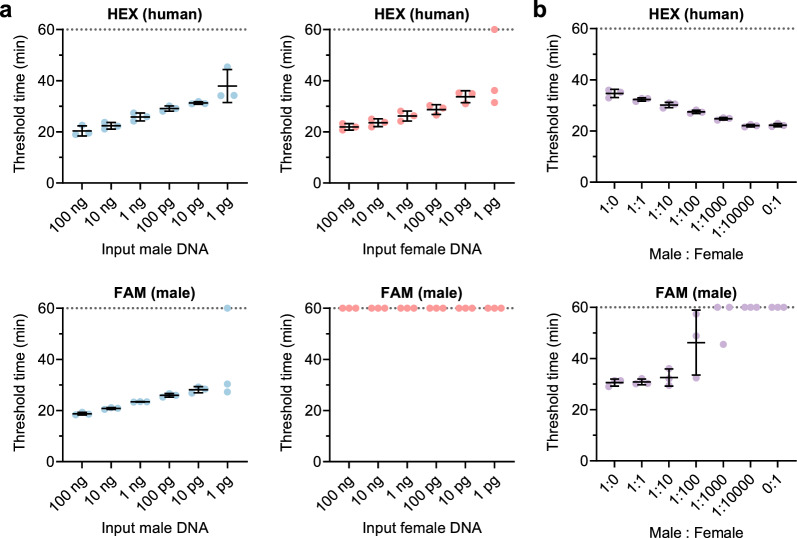
Performance of the duplex LAMP assay. **a** Sensitivity. Duplex LAMP assay was performed for tenfold serial dilutions (100 ng–1 pg) of the male and female DNA samples. **b** Mixed DNA samples. Duplex LAMP assay was performed for mixed DNA samples with male:female ratios of 1:1 to 1:10,000 (male DNA = 10 pg). The samples with ratios of 1:0 and 0:1 were the male DNA (10 pg) and female DNA (100 ng), respectively. **a**, **b** Data are expressed as mean ± SD (n = 3). Data at a threshold time of 60 min represent samples that were not detected

To determine the assay sensitivity, we assessed diluted control DNA samples. Our assay demonstrated robust detection of both the HEX (human target) and FAM (male target) signals from as low as 10 pg of the control DNA samples, with partial detection from 1 pg of the DNA samples (Fig. 3a).

### Mixed DNA samples

To examine the effect of female DNA on the detection of the FAM (male target) signal, we tested mixed DNA samples. Our assay successfully detected the FAM (male target) signal in the mixed samples with the male:female ratios ranging from 1:1 to 1:100 (Fig. 3b). However, the presence of 1000-fold excess female DNA inhibited the detection of the FAM (male target) signal. Because the HEX (human target) signal was generated faster with increasing the mixture ratio (Fig. 3b), we reasoned that the human-target LAMP product inhibited the male-target LAMP reaction. We confirmed this hypothesis by testing the male-target LAMP assay spiked with the human-target LAMP product (Additional file 1: Fig. S1).

### Body fluid samples

We tested the duplex LAMP assay on the DNA samples extracted from blood, saliva, semen, and vaginal secretions. Our assay correctly detected the HEX (human target) signal from all the tested samples and the FAM (male target) signal from only the male samples (Table 1).

**Table 1:** Analysis of body fluid samples

Sample (individual #)	Threshold time (min)	
	HEX (human)	FAM (male)
Male blood #1	31.09	26.97
Male blood #2	32.62	26.65
Male blood #3	31.50	27.63
Female blood #1	36.18	ND
Female blood #2	27.17	ND
Female blood #3	27.91	ND
Male saliva #1	29.31	28.02
Male saliva #2	29.81	29.18
Male saliva #3	30.30	30.06
Female saliva #1	29.17	ND
Female saliva #2	29.19	ND
Female saliva #3	29.13	ND
Semen #1	27.48	24.19
Semen #2	28.71	25.45
Semen #3	26.78	25.07
Vaginal secretions #1	20.97	ND
Vaginal secretions #2	21.85	ND
Vaginal secretions #3	21.02	ND

*ND* not detected

## Discussion

In the present study, we developed a duplex LAMP assay capable of detecting human and human male DNA simultaneously (Fig. 1). Previous studies have already reported the LAMP assays for screening of these DNA targets [5–8, 14]; however, these approaches could detect only a single target per assay. Our duplex LAMP assay can streamline the screening process, generating the HEX and FAM signals in one assay corresponding to the human and human male DNA, respectively.

We first evaluated the amplification specificity by testing the male and female genomic DNA samples (Fig. 2). Compared with the hydrolysis probe-based qPCR assay that requires only four primers and two probes for the duplex reaction, our duplex LAMP assay requires ten primers and two assimilating probes (Additional file 2: Table S1). Increasing the number of primers generally leads to primer dimer formation, thereby raising the potential risk of false-positive amplification and detection. In our assay, primer dimers resulting from primer/primer interactions are not problematic, but those formed by assimilating probe/primer interactions may generate false-positive signals irrespective of the LAMP products. Nevertheless, no false-positive fluorescence was observed throughout this study. This indicates that primer dimers were not formed by the assimilating probe/primer combination, supporting the high specificity of our assay. We then examined the assay sensitivity and found that 10–1 pg of DNA samples were detectable (Fig. 3a). This sensitivity was comparable to that of the multiplex qPCR assay [1, 2]. This result suggests that our duplex LAMP assay can screen low-level DNA samples.

We subsequently examined the effect of female DNA and/or its amplified products on the detection of the male DNA target. The results showed that male DNA detection was inhibited by a 1:1000 (male:female) ratio of mixed samples, namely 1000-fold excess of female DNA (Fig. 3b). By contrast, the commercial qPCR assay can detect male DNA even in the presence of 200,000-fold excess of female DNA [2]. Thus, compared with the qPCR assay, our assay would be limited in its use for mixed samples. A previous study has shown that the presence of 10,000-fold excess female DNA did not inhibit male DNA detection by the single-plex LAMP assay [14]. Based on this finding, we reasoned that the inhibition of the FAM signal (male target) in the mixed sample was due to the human-target LAMP product, not due to female DNA itself. Indeed, the FAM signal (male target) detection by the single-plex LAMP assay was inhibited by the human-target LAMP product (Additional file 1: Fig. S1). Moreover, with increasing the mixture ratio, the human-target LAMP product was produced faster than the male-target LAMP product (Fig. 3b). It means that by the time the FAM signal began to rise, many human-target LAMP products were already present. To enhance the tolerability for mixed samples, further optimization for the reaction components would be required. Finally, we could correctly detect the human and male targets in forensically relevant body fluid samples (Table 1), demonstrating the applicability of our duplex LAMP assay to actual forensic samples.

The LAMP reaction can be detected by various platforms, including fluorescent signal [7–10, 14], color change of indicator dyes [8], or lateral flow strip [14]. Although the colorimetric assay is the simplest method, it is incompatible with the duplex LAMP assay. In this study, we evaluated the duplex LAMP assay based on fluorescent signals and therefore used a laboratory-based real-time PCR instrument. Given the advantages of the LAMP technique (e.g., isothermal amplification and inhibitor tolerability), future studies could extend the duplex LAMP assay to an on-site screening tool using a portable fluorescence device or lateral flow strip. Overall, these findings suggest that our duplex LAMP assay has the potential to be a promising screening tool for forensic applications.

## Limitations

As the duplex LAMP assay described here is a proof-of-concept model, extensive validation will be required: e.g., increasing the number of samples, analyzing various types of forensic samples, and investigating the compatibility with the DNA typing workflow. Moreover, further optimization of the primers, probes, or reaction components may be needed to improve the assay performance.

### Supplementary Information

**Additional file 1: Figure S1.** Human-target LAMP product inhibits male-target LAMP reaction.

**Additional file 2: Table S1.** Primer and probe.

## Data Availability

The datasets used and analyzed during the current study are available from the corresponding author on reasonable request.
