# Supplementary material for: Duplex loop-mediated isothermal amplification assay for simultaneous detection of human and human male DNA
Source: BMC Res Notes. 2023 Aug 22;16:180. doi: 10.1186/s13104-023-06464-2 (PMC10464483; doi:10.1186/s13104-023-06464-2)
Supplement: Supplementary file 1 — Additional file 1: Figure S1. Human-target LAMP product inhibits male-target LAMP reaction. [file 13104_2023_6464_MOESM1_ESM.pdf]

Supplementary Figure S1

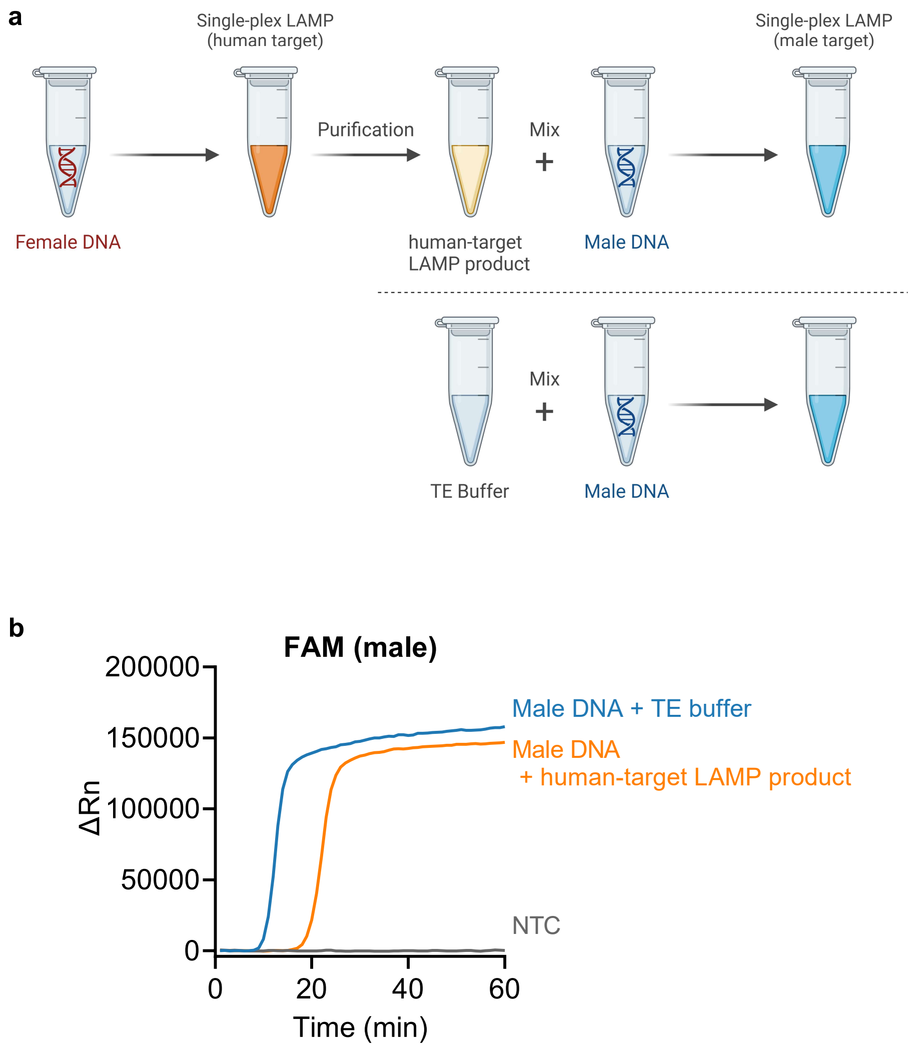

Fig. S1. Human-target LAMP product inhibits male-target LAMP reaction

a. Schematic representation of the experimental workflow. Initially, the single-plex human-target LAMP assay was performed for 10 ng of a female DNA sample. The reaction condition was the same as the duplex LAMP assay, with the following exceptions: 1 × Y-primer mix was excluded, and 1 × probe mix consisted of 0.16 µM HEX-HD-LB and 0.24 µM Quench strand. Subsequently, the human-target LAMP product was purified using the FastGene™ Gel/PCR Extraction Kit (Nippon Genetics, Tokyo, Japan) and eluted in 25 µL of nuclease-free water. Finally, the single-plex male-target LAMP assay was performed for 1 ng of a male DNA sample with or without the 5 µL of the human-target LAMP product. The reaction condition was the same as the duplex LAMP assay, with the following exceptions: 1 × HD-primer mix was excluded, and 1 × probe mix consisted of 0.08 µM FAM-Y-LF and 0.12 µM Quench strand. This figure was created with BioRender.com.

b. Representative amplification plots of the male-target LAMP assay.
